# Different types of uncertainty in multisensory perceptual decision making

**DOI:** 10.1098/rstb.2022.0349

**Published:** 2023-09-25

**Authors:** Stacey Aston, Marko Nardini, Ulrik Beierholm

**Affiliations:** Department of Psychology, Durham University, Durham, Durham DH1 3LE, UK

**Keywords:** decision-making, perceptual, multisensory, uncertainty, noise

## Abstract

Efficient decision-making requires accounting for sources of uncertainty (noise, or variability). Many studies have shown how the nervous system is able to account for perceptual uncertainty (noise, variability) that arises from limitations in its own abilities to encode perceptual stimuli. However, many other sources of uncertainty exist, reflecting for example variability in the behaviour of other agents or physical processes. Here we review previous studies on decision making under uncertainty as a function of the different types of uncertainty that the nervous system encounters, showing that noise that is intrinsic to the perceptual system can often be accounted for near-optimally (i.e. not statistically different from optimally), whereas accounting for other types of uncertainty can be much more challenging. As an example, we present a study in which participants made decisions about multisensory stimuli with both intrinsic (perceptual) and extrinsic (environmental) uncertainty and show that the nervous system accounts for these differently when making decisions: they account for internal uncertainty but under-account for external. Human perceptual systems may be well equipped to account for intrinsic (perceptual) uncertainty because, in principle, they have access to this. Accounting for external uncertainty is more challenging because this uncertainty must be learned.

This article is part of the theme issue ‘Decision and control processes in multisensory perception’.

## 1. Background

Choosing the best course of action depends on evaluating the evidence in favour of different options. A problem for any decision-maker is the quality of the data available. Major limitations to data quality come from the *bias* (systematic error) and *uncertainty* (random error, or noise or variability). An observer whose perception of the approach times of oncoming traffic is strongly biased, such that they systematically over-rate the available time to cross the road, is in danger. So is an observer whose perception is very uncertain: the high variability of their judgments means that, on any given occasion, there is a good chance of the percept deviating markedly from the true state.

An effective decision-maker should strive to minimize bias and uncertainty in the data on which they base their decisions. Here, we particularly consider the problem of uncertainty (noise). To preview our argument: (i) the data available for decision-making can include uncertainty (variability) for several reasons, and there is a key difference between intrinsic and extrinsic uncertainty; (ii) perceptual systems can be remarkably efficient at taking uncertainty into account, particularly via reliability-weighed combination of estimates, a noise-minimizing strategy; (iii) by contrast, people typically do not make such efficient decisions in tasks with uncertainty that is not perceptual; (iv) existing studies have explored ways in which perceptual and other tasks differ but have not clearly separated out the type of uncertainty alone as a factor; (v) we consider the possibility that ‘extrinsic’ uncertainty, of the kind common to non-perceptual decision problems, is more difficult to account for than ‘intrinsic’ uncertainty. To test this idea, we assess how people perform on a new spatial localization task, in which all decisions are made within the perceptual domain, and all use the same stimuli, but we manipulate the degree of uncertainty from intrinsic (perceptual) versus extrinsic (environmental) sources; and (vi) our finding is that perceptual judgments are indeed less effective in taking extrinsic uncertainty into account than intrinsic uncertainty. This illustrates that not all kinds of uncertainty are treated in the same way: internal uncertainty can in principle be accessible to perceptual systems, while external uncertainty needs to be learned.

### (a) Sources of uncertainty during perceptual decision making

Why are estimates uncertain (noisy, variable, limited in precision)? In the perceptual domain, limitations in sensory apparatus and computational imprecisions in the brain mean that sensory signals are subject to noise [1]. In perception, this noise becomes particularly evident when fine discriminations are required: a person may struggle to correctly select the biggest of two similarly sized apples, or the shortest of two similarly long supermarket lines. This uncertainty greatly increases when conditions for the sensory apparatus are degraded—for example, for a person choosing an apple in near darkness, or judging the supermarket line using eyes affected by progressive vision loss. We term this kind of uncertainty, related to variability (noise) in the sensory signal, *intrinsic—*in the sense that it is internal to the workings of a particular sensory system. We consider our example environmental (e.g. dark) or medical (e.g. vision loss) factors also to contribute to *intrinsic* uncertainty, as they lead to high variability (noise) in a particular sensory system. An ideal observer (or a different organism, or robot) equipped with better light-gathering apparatus would be subject to less noise during the nocturnal apple-selecting task.^1^ Intrinsic uncertainty reflects the variability (noise) within a particular sensory system, not variability (noise) in the environment.

By contrast, *extrinsic* uncertainty arises from variability in an environment, not in a sensory system. An example would be the variability in different lines' speeds at supermarket checkouts owing to the differing behaviours of individual cashiers. Cashiers’ speeds may vary as a function of their visuo-motor speed, their propensity to take time talking to customers, their familiarity with unusual items that otherwise need to be looked up, and so on. Excluding (in this example) reliable instantaneous perceptual cues to all the determinants of how fast a cashier is, the ideal observer could only establish relative speeds by observing the environment for some time. This process may *also* be limited by variability in the observer's perceptual processes—but crucially, even an ideal observer with negligible errors or computational limitations affecting these processes would still be subject to this externally determined uncertainty, and would need to spend time sampling the environment to build up an estimate of this source of variability—unless they had evolved, or been programmed to, have this information.

To sum up, we define intrinsic uncertainty as straightforwardly perceptual, in that it reflects the limitations of the perceptual process. Perception always comes with some uncertainty [1], but this uncertainty is particularly evident when making a fine discrimination and/or in degraded or sub-ideal conditions. By contrast, extrinsic uncertainty is outside of the perceptual process and reflects variability in the world itself. Examples include patterns of stochastic behaviour of agents or physical processes—e.g. the spread of droplets from a flow of water, the shooting accuracy of an archer, the probable waiting time for a bus. The statistics of these kinds of externally determined distributions can be learned, but the ideal observer needs to collect observations to learn them.^2^ Internal uncertainty within a perceptual process itself could, in principle, be much more readily available to the perceiver.

### (b) Efficient mitigation of intrinsic uncertainty via combination of estimates during perceptual tasks

A useful strategy for reducing uncertainty is combining a perceptual estimate either with another estimate of the same property [2,3], or with prior knowledge of the statistics of the environment [4]. This has the effect of averaging-out random noise [2]. In the perceptual domain, this process has been studied in *sensory cue combination* tasks, which measure people's abilities to reduce the uncertainty of a sensory estimate by combining it with other available estimates [5]. In a classic study [6], people judged which of two bars was taller, using vision and/or touch. The ideal observer would obtain predictable reductions in their uncertainty given both estimates together versus either alone by reliability-weighted averaging—in which each estimate is weighted in inverse proportion to its uncertainty [5]. Participants followed this ‘optimal’ strategy: they obtained the theoretically maximum uncertainty reduction when given the opportunity to combine cues, and they re-weighted (changed their relative reliance on) the visual cue to height as it was made more uncertain by addition of stereo noise [6]. Similarly, participants use prior statistical information to reduce their perceptual uncertainty, for example predicting visually noisy trajectories based on statistical distributions learned over the course of the experiment [4]. Observers often near-optimally combine noisy sensory estimates with each other [6–9] and with prior distributions [4,10–12], although there are also cases of suboptimal combination [13–15]. Perceptual cue combination is already evident in single neurons of early sensory areas during multisensory tasks [16], and can also be detected using functional magnetic reasonance imaging (fMRI) in humans, in sensory areas (even during passing viewing, in the case of combination of visual cues to depth) [17–19] and across a whole cortical hierarchy up to the frontal lobe during perceptual decision tasks [20–22].

For this kind of reliability-weighting to work, the perceptual system must correctly account for its own uncertainty. For example, it must adjust the weighting given to a visual cue to bar height, versus a haptic one, in line with more or less stereo noise being added [6]. Similarly, during visual-auditory localization [7], the observer must adjust the weighting given to a visual cue to location, versus an auditory one, in line with progressive visual blurring. How uncertainty-weighted averaging is implemented at a cells-and-circuits level is still being established [23–25], but it is clear that uncertainty intrinsic to perceptual estimates can be represented implicitly by population responses, because elementary perceptual properties tend to be represented by populations of tuned cells. Thus, a very precise estimate of a line's orientation might strongly engage a relatively small population of cells tuned to a specific orientation, while an uncertain estimate of orientation, e.g. in a more blurred stimulus, would more weakly engage a broader population. The distribution of population responses can therefore reflect the level of intrinsic uncertainty. Consistent with this, widths of neuronal probability distributions decoded from participants' fMRI activity are predictive of their perceptual decisions [26,27].

In summary, perceptual tasks show abilities to account effectively for perceptual uncertainty. When sensory estimates are combined [6–9], all the uncertainty is within the perceptual system itself (tasks combining sensory information and priors [4,10–12] have the additional challenge of learning and representing the prior). It is not obvious that perceptual systems should necessarily accurately read out or represent the variabilities in their own estimates, but the evidence from cue combination tasks [6–8] suggests that this must be the case. Considering how perceptual information is represented by neuronal populations [23–27] also provides plausible mechanisms for representing and reading out this variability information within the perceptual system itself, without the need to additionally learn about this variability.

### (c) Less efficient mitigation of uncertainty during non-perceptual decision tasks

The literature on economic decision making under uncertainty stands in stark contrast to these findings from perceptual tasks, documenting many inefficient and seemingly sub-optimal patterns of decision making [28–31]. By ‘classical’ decision making, we mean decisions about verbally or symbolically presented options. One example of this type of problem is a lottery, such as ‘would you rather gain $3000 for sure or have an 80% chance of gaining $4000?’ [29]. A second is a decision informed by a *base rate* (similar to a *prior* in perceptual tasks), for example: how likely it is that a blue versus green taxi was responsible for a traffic accident, knowing that 85% of taxis in the city are green and 15% blue, and that the witness (who judged that it was blue) is only correct at making this discrimination 80% of the time? (the ‘taxicab problem’ [30]). A third kind is a multiple-factor combination judgement, such as deciding how toxic a fictitious bug is likely to be based on multiple predictive cues (e.g. leg length, colour) [31].

Perhaps most pertinent to the questions asked in this study is the issue of weighting of experience versus externally provided statistics. A veteran physician will have accumulated years of experience of diagnosing common diseases, but may have no experience with very rare diseases. A possible problem is that they may rely too heavily on their experience, and under-value the potential of diseases not previously diagnosed, possibly owing to the representativeness heuristic [32]. (This is in contrast to a more junior physician who might over-represent the possibility of diseases that are very rare, an example of base rate neglect [33].) Phrased in terms of Bayesian inference, the experienced diagnostician might put too little weight on a likelihood of a disease that is verbally/symbolically presented to them, instead putting too much weight on their prior experience. Formally, this can also be used to describe the cause of stereotyping [34].

Tasks and situations like these are non-perceptual in the sense that the information (e.g. words, numbers, bug features, disease prevalences) is symbolic and not subject to relevant perceptual uncertainty. They can also differ from the perceptual tasks described above in the specific information integration problem (in the cue combination examples and in the taxi-cab and bug problems, multiplying likelihoods, probability distributions or probabilities; in the lottery example, combining posterior probability and value). Our aim here is to focus specifically on the source and representation of uncertainty, and to bring these kinds of problems closer, leading us to introduce a novel experimental task in which every aspect of the task except for the type of uncertainty is matched. In order to reach that point, we first review previous studies in which aspects of perceptual and non-perceptual decision tasks have been matched and compared in significant ways.

### (d) Task differences and uncertainty differences

Studies have directly compared people's abilities to deal with internal estimates of uncertainty on the one hand, and explicitly stated probability information on the other. Some studies measured participants' visuomotor precision at a speeded pointing task [35,36], allowing them to calibrate the visual stimuli so that the same verbal lottery could equivalently be presented as a visuo-motor one. In the verbal task, participants had to choose which of two numerically expressed probabilistic scenarios they would prefer (of the general type x% chance of winning A versus y% chance of winning B), while in the visuo-motor task, they made equivalent decisions by choosing which of two visual targets they would prefer to attempt to hit for their respective rewards. One study found different biases towards risk-seeking and different weighting of probability and value across these tasks [35]; another found broadly comparable performance [36]. This general approach compares intrinsic (visuo-motor) and extrinsic (stochastic) uncertainty, but there are other differences in the two task types: visuo-motor uncertainty is presented perceptually by a bar width, while extrinsic (stochastic) uncertainty is given via the classical numeric (symbolic) route. Therefore, differences in behaviour may depend in part on the origin and nature of the uncertainty (internal versus external) and in part on the manner of presentation (perceptual versus symbolic).

A study wholly within the perceptual domain manipulated an array of oriented gratings in two ways [37]: by changes in the stimulus contrast, and changes in the variability of orientations across the different gratings in the array. Expressing this in our framework, reducing contrast increases intrinsic (perceptual) uncertainty, while increasing variability increases the difficulty of the information integration problem of correctly averaging together disparate estimates. This study did not have ‘extrinsic’ uncertainty (nothing is stochastic: an ideal observer without limitations in perceptual precision or computational abilities could deal with both the contrast and the variability manipulations), but is related by the authors to cognitive decision-making in an interesting way. They found that participants took contrast-related (intrinsic) uncertainty near-optimally into account, but failed to do so for variability-related (integration-demanding) uncertainty, and were well described by a model blind to this source of noise. The authors propose that the ‘optimality gap’ in perceptual versus classical cognitive decision tasks may be explained in part by a blindness to noise introduced during the integration process that combines multiple information sources. The argument is that even perceptually presented estimates (low-contrast grating orientations), when combined in a non-standard way for the sensory system (averaging multiple gratings in an array), become subject to cognitive-like decision errors. Presumably, the contrast is between this type of novel averaging and the highly familiar averaging during cue combination where multiple sensory cues are associated with a single redundant property, such as size [6] or location [7]. This study interestingly bridges perceptual uncertainty and the integration/decision process, suggesting a privileged role for intrinsic (perceptual) uncertainty and a relative blindness to noise that affects a computational (averaging) process.

A recent study took a different approach to comparing how people presented with cognitive inference tasks approach the sort of precision weighting of estimates common in perceptual cue combination [38]. Participants performed three different cognitive decision tasks that require combining prior information with new data, and a model-based analysis was used to categorize them according to the extent to which they followed different principles underlying optimal inference. Just over half of participants appreciated the need to consider the prior as well as the data, but only a quarter appreciated the need to weight them according to their uncertainties. While there is no direct comparison with the perceptual domains within this study, and perceptual studies rarely categorize individual subjects in this way (though see [39]), this very widespread blindness to uncertainty weighting during cognitive tasks is at odds with its usual strong implementation during perceptual tasks [4,6–12].

In summary, while perceptual decision making in the face of perceptual (intrinsic) uncertainty is often highly efficient in dealing with this uncertainty [4,6–12], this is not typically seen in classical or cognitive decision making [28–31,35,38]. A crucial question is to what extent different systems and representations underlie decision making in these domains. However, these domains differ across many dimensions. Examples of ways in which studies have explored these differences include whether probabilistic information is presented perceptually or symbolically [35,36], how much uncertainty is at a perceptual versus at an integration stage [37], and which models best explain precision-weighting behaviour during cognitive tasks [38].

Behaviour during decision-making under uncertainty may differ for many of these and other reasons, but here we return to the key distinction introduced at the start: the source and nature of the uncertainty itself. It has been widely appreciated that internal uncertainty, owing to variability within the perceptual system [1], and potentially amenable to being read out by the system itself [23–27], has advantages compared with external uncertainty, which depends on stochastic events outside of the observer. It has also been appreciated that a privileged access to internal uncertainty may underlie many perceptual-cognitive task differences. However, tasks probing these differences tend not to allow for a clean interpretation of the role of type of uncertainty alone in judgments—since these also present the information in different ways [35,36], or do not compare these two types of uncertainty [37,38].

### (e) Explaining task differences: the present study

It may be that different systems and representations underlie decision making across perceptual and cognitive domains and that the relative engagement of these depends on a range of task, informational and perceptual parameters. However, here we consider one crucial factor that has not been clearly distinguished and tested: the nature of the uncertainty involved, intrinsic versus extrinsic.

In the present study, we directly compare abilities to account for intrinsic versus extrinsic uncertainty during a perceptual decision-making task. Crucially, the task and even the stimuli are exactly matched across conditions, so that uncertainty is signalled in the same way (via spread of dots), and decisions and responses are made about the same property (location) and entered in the same way (clicking to indicate a screen location). We use a multisensory localization task, in which visual (dot cloud) and auditory (white noise on a co-localized multi-speaker setup) provide redundant cues to the property to be estimated: location. We draw the dot clouds such that we calibrate the amount of noise that is intrinsic (reflecting sensory imprecision at determining the centre of the cloud^3^) versus extrinsic (reflecting unreliability of the cloud centre as a guide to the target location). The colours of the dot clouds, together with a cover story (that they are guesses by two different players) are the cue to which levels of intrinsic and extrinsic noise underly a given trial. Since intrinsic uncertainty is available to the perceptual system itself, while extrinsic must be learned with experience, we predict that decision-making in the intrinsic case will show better following of signal reliabilities.

### (f) Preview of findings

We directly compared abilities to account for intrinsic versus extrinsic uncertainty during a perceptual decision-making task. Our main finding was near-optimal weighting of sensory estimates when uncertainty was only intrinsic, but mis-weighting of estimates when extrinsic uncertainty was added. Specifically, participants over-relied on an extrinsically uncertain signal, suggesting that they are to some degree ‘blind’ to this source of uncertainty. This illustrates that not all kinds of uncertainty are treated in the same way: internal uncertainty may be accessible to the perceptual system, while external uncertainty needs to be learned.

## 2. Methods

Thirty participants (26 female, min/median/max age 18/24/34 years, age information for one participant was lost) completed a series of trials where they used intrinsic+extrinsic visual cues, intrinsic-only visual cues and intrinsic-only auditory cues to estimate the location of a hidden target on a projector screen.

Participants were recruited from a combination of undergraduate students in the Psychology department and social media, and were compensated for their time (£10 per hour plus performance bonus). The only restrictions were corrected or uncorrected normal vision and normal hearing. Participants were fully briefed about the purpose of the experiment and gave informed consent, with ethical approval given by Durham Psychology department.

Participants were placed 140 cm in front of a projector screen (width 235 cm, height 131 cm), with all visual stimuli projected onto it (Optoma GT1080E). Behind the screen were placed nine speakers (Visaton SC 5.9) at a separation of 2.5 visual degrees.

As a cover story, participants were told that they were taking part in a task similar to a fairground game, where they had to find a hidden object using visual cues of previous players, possibly together with the rustling sound made by the person placing the hidden object (see the electronic supplementary material for the full instructions). For the two types of visual stimuli (intrinsic-only and intrinsic+extrinsic, see below) participants were told the cues were guesses by two different individual players, ‘Rodger’ and ‘June’, signalled by different coloured dots (blue or orange). The exact instructions given to participants are included in the electronic supplementary material.

**Figure 1.**
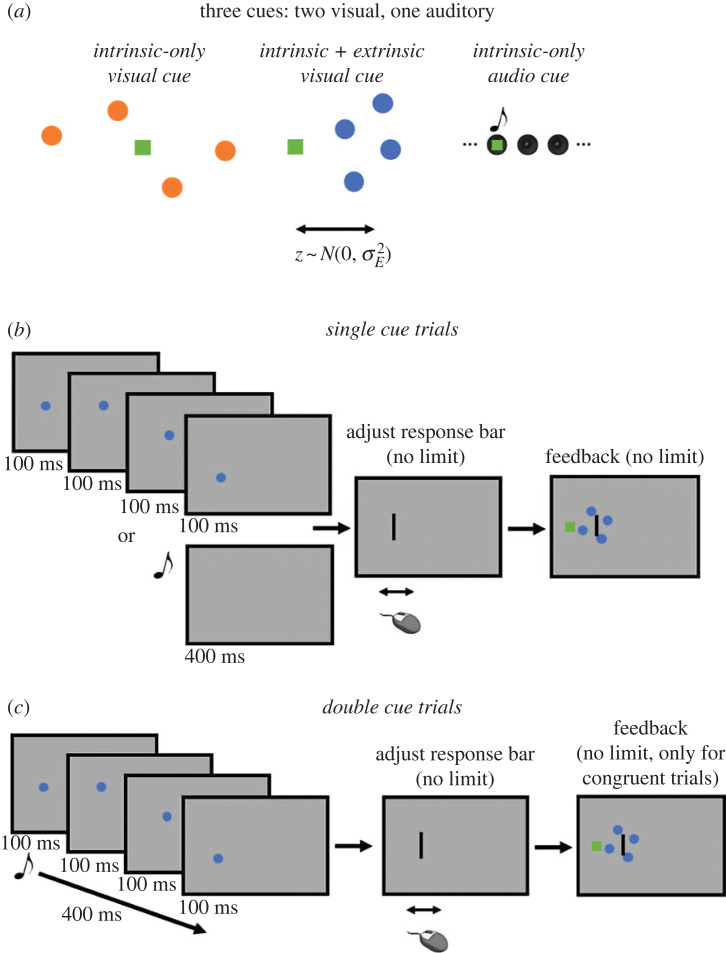
Schematics of experiment. (*a*) Subjects were presented with three different types of cues: intrinsic-only visual, intrinsic+extrinsic visual, intrinsic-only auditory. The total variance of the intrinsic+extrinsic cue (blue) was matched to the intrinsic only visual cue (orange) by adding noise to the mean of the four dots. (*b*) Subjects on some trials were presented with single cue stimuli of either the visual or auditory cue. Dots were presented sequentially, while the auditory cue was continuous for 400 ms. Feedback was provided for all single cue trials. (*c*) For on other trials, subjects could be presented with two cues (visual and auditory). The cues were identical to the single cue trials, but feedback was only provided on trials where visual and auditory cues were congruent (i.e. from the same location).

The experiment began with a visual cue calibration block where participants estimated the location of a hidden target using low or high variance intrinsic-only visual cues (four-dot clouds, each dot shown in succession for 100 ms, generated by shifting and scaling the four dot centres drawn from a standard normal distribution so that the mean was exactly the true location, and the standard deviation (s.d.) was fixed at 0.05 or 0.2, respectively; stimulus parameters are defined as proportions of total screen width such that zero maps to the left of the screen and one to the right). There were 90 interleaved trials for each cue (10 repeats for nine test locations; approximately evenly spaced points between 0.37 and 0.63). We interpret dot clouds generated in this way as being corrupted only by intrinsic noise as variability in location estimates made using these cues is owing to noise that is intrinsic to the observer, such as sensory noise. Put differently, an ideal observer who is free from sensory noise, computational imprecision, memory imperfections, response noise, etc. could provide a perfect estimate on every trial (figure 1*a*).

The purpose of the initial visual cue calibration block was to measure perceptual variance using each of the low and high variance intrinsic-only visual cues ($\sigma_{L}^{2}$ and $\sigma_{H}^{2}$, respectively) so that the difference in variance using each of the two cues ($\sigma_{E}^{2}=\;\sigma_{H}^{2}-\sigma_{L}^{2}$) could be used as the variance of the extrinsic noise distribution. The extrinsic noise distribution was used to generate random shifts ($z_{i}∼N(0,\sigma_{E}^{2})$, where $z_{i}$ is independently drawn for every appearance of each intrinsic+extrinsic sensory cue) of all dots in a cloud (same shift for each dot in a single cloud, or equivalently, only one $z_{i}$ per trial) away from the true hidden location. For the remainder of the experiment, extrinsic noise generated in this way was only added to the low intrinsic-only cue to create a single intrinsic+extrinsic visual cue. Adding noise in this way, with the level of noise calibrated individually for each participant, should lead to participants being equally variable using the intrinsic+extrinsic visual cue and the high intrinsic-only visual cue (referred to hereafter as the intrinsic-only visual cue). Indeed, that was our intention to allow for a direct comparison of the weight placed on each cue.

The visual cue calibration block was followed by the audio cue training block, where participants used an intrinsic-only auditory cue to estimate the location of the hidden object. The intrinsic-only auditory cue was a 400 ms burst of white noise from one of nine speakers located behind the projector screen presenting the dot clouds. The locations corresponded to the locations used in the calibration block and we tested each location 10 times for a total of 90 interleaved trials in this block. We say this auditory cue is only intrinsically uncertain as an ideal observer, who perfectly localizes sounds, would choose the exact location of the hidden object on every trial. Thus, variability in the responses is intrinsic to the observer, again generated by factors such as sensory noise, computational imprecision and response noise.

The audio cue training block was followed by the test block. In the test block of the experiment, participants used either the intrinsic+extrinsic visual cue, intrinsic-only visual cue, or an intrinsic-only auditory cue alone to estimate the hidden object location (single cue trials; figure 1*b*), or one of the visual cues paired with the auditory cue (double cue trials; figure 1*c*). In total, there were 405 single cue trials (figure 1*b*) in the test block, made up of 15 repeats of each location used in the calibration block for each single cue (intrinsic+extrinsic visual cue, intrinsic-only visual cue, and intrinsic-only auditory cue). The single cue trials were interleaved with congruent and incongruent double cue trials (figure 1*c*). The intrinsic-only and intrinsic+extrinsic trial types were randomly interleaved. In congruent double cue trials, each visual cue could be presented simultaneously with the auditory cue with both cues corresponding to the same hidden location. There were 270 congruent double cue trials made up of 15 repeats of each location used in the calibration block for each audio-visual cue pairing. Incongruent double cue trials were like the congruent cue trials, with each visual cue being presented simultaneously with the auditory cue, except that in these trials, rather than indicating the same location, these cues were in conflict, allowing us to estimate the amount of weight that participants placed on each visual cue when paired with the audio cue. There were 400 incongruent double cue trials, made up of 10 repeats of all combinations of intermittent calibration locations (0.37, 0.43, 0.5, 0.57, and 0.63) for each audio-visual cue pairing.

Owing to an error in the experimental code for the incongruent conditions of the test block, only stimuli in the left hemisphere were presented. To ensure there were no left-right biases which could affect our results we compared variable error in left versus right hemisphere for the intrinsic-only, as well as intrinsic+external, congruent conditions and found no significant difference (Wilcoxon signed-rank test, *p* = 0.8555, median difference = −0.0037 for congruent intrinsic only, *p* = 0.8555, median difference = 0.0011 for congruent intrinsic plus extrinsic). Encouraged by this we continued with the originally planned analysis (a post-hoc analysis for the incongruent data that allowed the central tendency bias to be shifted away from the screen centre found no differences, see the electronic supplementary material).

Participants received feedback on every trial in the visual cue calibration block, the audio cue training block, and on single cue and congruent double cue trials in the test block. Feedback consisted of a green circle or square that indicated the location of the hidden object, presentation of all dots from the dot cloud simultaneously on any trial involving a visual cue, and a score out of 1000. Feedback showing the object location alongside all four dots was the means by which participants could, in principle, learn the magnitude of the extrinsic noise on intrinsic+extrinsic trials (noting that two different signals, shown by differently coloured dots, indicated two individual named ‘players'—see participant instructions above and in the electronic supplementary material).

Scores followed a squared error loss function so that the score reduced quadratically with increasing distance from the target until it reached zero. The function was scaled so that the function would reach 0 points at a distance from the target of 10% of the screen width (0.1). With this modification, participants only score if the absolute difference between the hidden and guessed locations is less than 0.1. This may seem small, but as the experiment was completed on a projector screen of width 190 cm, this means participants only received points if their guess was within 19 cm of the true location on the screen. The formula for calculating the score was:

$$s=\;1000(1-{\left((h-g)/0.1\right)}^{2}),$$

where s is the score, h is the hidden location and *g* is the guessed location.

### (a) Calculating perceptual variance

During the experiment, we calculated variance using each of the low and high variance intrinsic-only visual cues in the calibration block as the standard deviation of all errors (response–target location). These values ($\sigma_{L}^{2}$ and $\sigma_{H}^{2}$) were used to define the variance of the extrinsic noise individually for each participant ($\sigma_{E}^{2}=\sigma_{H}^{2}-\sigma_{L}^{2}$) that was added to the low intrinsic-only visual cue to create the intrinsic+extrinsic visual cue. This was calculated online in the experiment immediately following the calibration block so that test trials could be generated. As mentioned above, adding extrinsic noise in this way should lead to equal variance when estimating the location of the hidden object using either the intrinsic+extrinsic cue or the intrinsic-only (high noise) cue in the test block of the experiment.

Since collecting these data, we have become aware of an important issue with variance estimates from data with continuous responses, such as those here: a frequent finding of central tendency biases [41–43], where participants bias their responses towards the middle of the set of presented stimuli. Such biases lead to an under-estimation of variable error. We have recently described a method that recovers corrected estimates of variable error [44] in this situation. As described in the electronic supplementary material and [44], we apply this method to recovering correct variable errors in the present dataset. Unfortunately, the variance calculations that were done online to calibrate stimuli within the experiment itself did not use this correction, and as a result we did not match variable error using the intrinsic-only and intrinsic+extrinsic visual cues in the test block as intended. Matching the conditions would allow us to have a simple non-model based comparison, and while the difference in variance makes the two conditions a little less comparable than intended, it does not prevent us from comparing the extent to which participants weighted stimuli appropriately in response to the two kinds of uncertainty using our model-based analysis. In short, given the corrected uni-modal variances it is straightforward to calculate the expected visual weighting and compare to the empirical measured weight.

### (b) Summary and predictions

In summary, we asked participants to localize a hidden object using visual (dot-cloud) and/or auditory (white noise) cues. The object's location was uncertain because of either only intrinsic noise (visual or auditory), or because of extrinsic as well as intrinsic noise. Extrinsic noise was implemented as an additional offset of cue dots versus the true location. Through feedback, participants had an opportunity to observe and learn about the level of this extrinsic noise.

Using trials with minor offsets in cue positions, we measured the relative weighting for (reliance on) visual versus auditory cues during perceptual judgements. We predicted that, in line with previous sensory cue combination studies [6–9], participants would not weight the visual cue with only intrinsic noise differently to the optimal (reliability-weighted) prediction. We predicted that, in contrast, participants would overweight the intrinsic+extrinsic cue, as they would be less sensitive to the added extrinsic uncertainty.

All stimuli, data and code are available at https://osf.io/6paq9/.

## 3. Results

### (a) Participants overweight the visual cue with extrinsic uncertainty

We used estimates of variable error using the intrinsic-only, intrinsic+extrinsic and auditory cues alone in the test block ($\sigma_{I},\sigma_{\mathrm{IE}}$ and $\sigma_{A}$) to calculate the optimal, reliability based, weight that participants should place on each cue in a pair. The formulas we used to calculate the optimal weight to place on the intrinsic-only cue ($w_{I}$) and the intrinsic+extrinsic cue ($w_{\mathrm{IE}}$) when either cue is paired with the auditory cue are given below.

$$w_{I}=\;\frac{\sigma_{A}^{2}}{\sigma_{I}^{2}+\sigma_{A}^{2}}\;w_{\mathrm{IE}}=\;\frac{\sigma_{A}^{2}}{\sigma_{\mathrm{IE}}^{2}+\sigma_{A}^{2}}.$$

This formulation is reliant on small discrepancies between visual and auditory stimuli (less than 10 visual degrees), for large discrepancies subjects might instead rely on a causal inference model [40,45].

The empirical weights were determined by modelling responses to the incongruent trials as

$$r=(1-{\hat{w}}_{P})({\hat{w}}_{V}x_{V}+{\hat{w}}_{A}x_{A})+0.5{\hat{w}}_{P}+\epsilon ,$$

where ${\hat{w}}_{P}\;$estimates the strength of the central tendency bias (for this cue pairing), ${\hat{w}}_{V}$ estimates the weight placed on the visual cue (the intrinsic-only or intrinsic+extrinsic cue: ${\hat{w}}_{I}$ or ${\hat{w}}_{IE}$), ${\hat{w}}_{A}=(1-{\hat{w}}_{V})$ estimates the weight placed on the auditory cue, $x_{v}$ is the centroid of the dot cloud (the same as the visual cue source location for the intrinsic-only cue but not for the intrinsic+extrinsic cue), $x_{a}$ is the source location of the auditory cue, and $\epsilon ∼N(0,\sigma_{n}^{2})$ is a noise term.

To allow for the fact that we assume subjects receive a noisy (uncertain) input, the perceptual cues had noise with variance of $\sigma_{V}^{2}$ and $\sigma_{A}^{2}$, with ${\hat{w}}_{V}=\sigma_{A}^{2}/(\sigma_{A}^{2}+\sigma_{V}^{2})$. A participant with a veridical estimate of their own relative perceptual uncertainties would thus correctly set their variances based on these. For a linear model this has no effect on the mean estimates, but can affect estimates for more complicated models.

We fitted this model separately for each participant to all conflict trial responses for combinations of the intrinsic-only and auditory cue, and intrinsic+extrinsic and auditory cue separately. The model was fitted using JAGS [46] via the MATLAB-to-JAGS interface matjags.m to estimate posterior probability distributions for the free parameters ${\hat{w}}_{P},{\hat{w}}_{V},$ and $\sigma_{n}$.

We ran three independent chains, discarding the first 1000 samples of each chain as burn-in, and recording 4000 samples after the burn-in period, thinned by recording only every fifth sample. Both fitted weights (${\hat{w}}_{P}$ and ${\hat{w}}_{V}$) were initialized at 0.5 in all chains. The standard deviation of the noise ($\sigma_{n}$) was initialized at 0.01. The priors on $w_{V}$ and $w_{p}$ were uniform distributions between 0 and 1. The prior on $\sigma_{n}$ was a uniform distribution between 0.001 and 0.2. The final parameter estimates were taken as the mean of the expected values for each chain.

**Figure 2.**
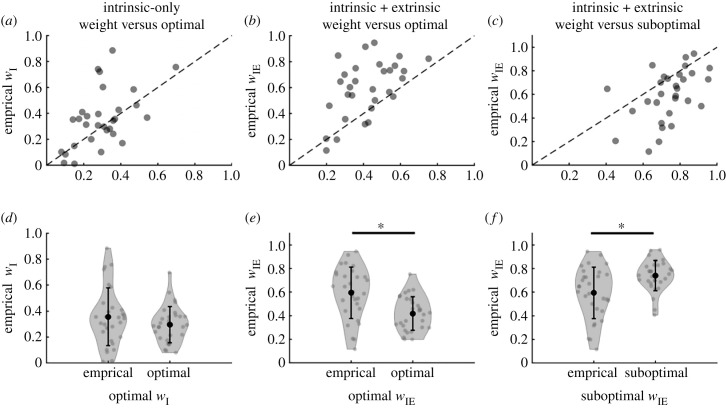
Comparison between visual weights for intrinsic-only (*a*) and intrinsic-extrinsic (*b,c*), empirically measured weights against either optimal weights (*a,b*) and suboptimal insensitive weights (*c*). While both intrinsic-only and intrinsic + extrinsic empirical weights correlated with the optimal weights, the visual weights for intrinsic-only were not significantly higher than optimal (*d*), whereas the weights for intrinsic + extrinsic were significantly higher than optimal (*e*). Intrinsic+extrinsic visual weights were also significantly lower than weights from a suboptimal model that ignores the extrinsic variability (*f*).

Figure 2*a* shows that the weight participants placed on the intrinsic-only cue relative to the auditory cue is positively correlated with the optimal prediction (r=0.536, p=0.002), suggesting participants weight the cues according to their reliabilities. Figure 2*d* shows that empirical and optimal weights do not differ significantly ($t_{29}=-1.74,\;p=0.092$), although the Bayes factor (BF) in favour of the null is approximately 1, suggesting both the null and alternative hypotheses are equally good explanations of the data (BF01 = 1.34).

Figure 2*b* shows that the weight participants placed on the intrinsic+extrinsic cue relative to the auditory cue is also positively correlated with the optimal prediction (r=0.524, p=0.003), again suggesting participants weight the cues according to their reliabilities. However, most points are above the identity line (empirical weight greater than optimal). Figure 2*e* shows that mean empirical and optimal weights differ significantly ($t_{29}=\;-5.14,\;p<0.001$), with participants overweighting the intrinsic+extrinsic visual cue. The BF in favour of the alternative suggests there is extreme evidence for a difference between empirical and optimal weights (BF10=1291.3).

We hypothesised that participants would overweight the intrinsic+extrinsic cue as they would be insensitive to the added extrinsic uncertainty. If participants are completely insensitive to the extrinsic uncertainty, they should weight the intrinsic+extrinsic cue according to the variability of their responses when using the low intrinsic-only cue measured in the calibration block. We call the use of this insensitive strategy the suboptimal intrinsic+extrinsic cue weight prediction. Figure 2*c* shows that empirical weights are correlated with the suboptimal insensitive predictions (r= 0.482, p=0.007), but the position of most points below the identity line and figure 2*f* show that empirical weights differed significantly from the suboptimal insensitive predictions ($t_{29}=\;4.16,\;p<0.001$). The BF in favour of the alternative suggests there is extreme evidence for a difference between empirical and suboptimal weights (BF10=110.7). This suggests that participants are not completely insensitive to the extrinsic uncertainty, but only partially account for it.

### (b) Individual differences

**Figure 3.**
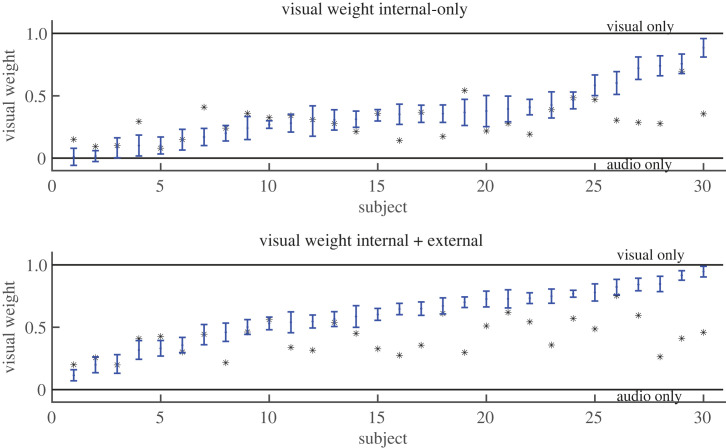
Individual visual weights, with 2* standard deviation error bars, as reported from JAGS Bayesian fit to the intrinsic-only (above) and intrinsic+extrinsic (below) datasets. Grey asterisk indicates the optimal weighting. A value of 0 indicates all weight on audio, a value of 1 that all weight is on visual.

An advantage of running a Bayesian model with JAGS is that it provides uncertainties around the variable fits, unlike a maximum likelihood approach which just gives the best fitted parameter value. We can therefore also examine how well individual subject parameters were fitted, e.g. the Bayesian confidence intervals of parameters such as the visual weight. Using the JAGS output we calculated the Bayesian confidence interval for the visual weight, finding that for the intrinsic-only dataset for 18 out of 30 participants the optimal weight was outside their 95% confidence intervals, while for the intrinsic+extrinsic dataset the proportion was 22 of 30 participants (figure 3).

To explore further what participants did we also compared the empirically fitted weight values to those of a model participant who only used their best modality (whether visual or auditory). Only two of the participants in the intrinsic-only condition had a Bayesian confidence interval that included the possibility of just using their best modality, and none of the participants did in the intrinsic+extrinsic condition. For all the remaining participants (28 and 30) we could therefore rule out that they only used their best modality.

### (c) Switching

One possible reason that participants could attain a weight on the visual cue that would be indistinguishable from optimal would be to perform a random switching between reporting the noisy percept from the two modalities, at the right ratio but without actually combining them (also referred to as probability matching). Such behaviour has previously been found in perceptual cue integration experiments (e.g. [47]). To rule out this idea we ran a variant of the model above that included an individually fitted probability of the participants performing switching instead of integration. We found a low average probability of subjects using a switching strategy of 0.195 for intrinsic-only and 0.212 for intrinsic+extrinsic. These values were not significantly different (rank sum test *p* = 0.631), hence there was no evidence that the datasets differed based on propensity to use switching over integration. Overall, this implies that there is a small likelihood that participants were using a switching strategy and that it was more likely that were indeed combining stimuli based on the optimal weights for the intrinsic-only dataset (but not for intrinsic + extrinsic).

### (d) Variable error

An advantage of using Bayesian inference is the reduction in error, hence if we expected participants to be using optimal weighting there should be an advantage in error. However in practice these error reductions are only found when visual and auditory stimuli are calibrated to have well matched error levels, otherwise the ideal observer predicted advantage may differ too little from the best single cue to be measurable in noisy responses [48,49]. Unfortunately, as we did not foresee the issue of central tendency bias (see Methods above, [44], and the electronic supplementary material), we did not match variable error using the intrinsic-only and intrinsic+extrinsic visual cues in the test block as intended (see the electronic supplementary material).

The variable error for the intrinsic-only dataset (calculated for congruent audio and visual stimuli, unlike the calculation above for visual weight based on incongruent stimuli) did not show a significant improvement over the variable error for the single best modality for each participant (Wilcoxon signed-rank test, *p* = 0.371, median = −0.002). Non-intuitively, there was however a small but significant advantage of using intrinsic + extrinsic visual together with audio (*p* = 0.032, median = 0.004), probably owing to the better matching of the visual error with the audio error. The unintended higher uncertainty in the intrinsic only than the intrinsic+extrinsic condition meant that noise in this condition was less well matched to the audio noise, leading to a lower potential gain. See the electronic supplementary material for full details of this analysis.

## 4. Discussion

We introduced a distinction between intrinsic and extrinsic uncertainty during decision-making. While perceptual and other decision tasks can vary in many ways, a key difference is often the kind of uncertainty involved. Perceptual decision-making usually involves uncertainty that is intrinsic to the perceptual system itself, and to which it may therefore have good access [23–27]. In line with this, the intrinsic uncertainty in perceptual tasks is often efficiently mitigated by reliability-weighted combination [6–9]. By contrast, when uncertainty arises in processes external to the observer, it must be learned in order to be accounted for. While this difference may go some way towards accounting for differences in how people deal with uncertainty across decision tasks, those tasks typically have many other differences too. Therefore, in the present study, we developed a perceptual task to make this specific comparison directly. To do so, we manipulated levels of intrinsic versus extrinsic uncertainty while keeping the task and stimuli the same across these manipulations. We tested the extent to which decision-makers take uncertainty into account when using multiple sensory estimates for a localization task, and tested the prediction that participants would follow the reliabilities of estimates better with intrinsic-only than with intrinsic+extrinsic uncertainty.

As predicted, we found near-optimal (or rather, not statistically different from optimal) weighting of sensory estimates when uncertainty was only intrinsic, but mis-weighting of estimates when extrinsic uncertainty was added. Specifically, participants over-relied on an extrinsically uncertain signal, suggesting that they are to some degree ‘blind’ to this source of uncertainty. However, they relied on it more than the prediction for participants completely blind to the extrinsic uncertainty. That is, participants fully accounted for uncertainty when it was purely intrinsic, but only partially accounted for it when it was also extrinsic. These results are in line with the observation that intrinsic uncertainty within a perceptual process can in principle be read out from the estimate's neural representation [23–27], while extrinsic uncertainty as reflected by the statistics of how an external process or agent behaves needs to be learned—either from an external information source [29–31], or (as here), by directly learning from experience.

These results also add to a growing literature on the description-experience gap [50], which shows how information that is not directly experienced, but based on abstract symbolic representation, is not correctly weighted within a Bayesian framework. This account of the issue of an experienced physician under-weighting the likelihood of a disease they have not personally encountered (see Introduction) would propose that they do not accurately represent the uncertainty of this information, as it was not experienced but merely described to them. In our task, the new information about external uncertainty was experienced, but was presented and learned in a manner different to internal noise. This provides a clue to which factors are important for the correct Bayesian inference: an insight is that not only whether the information is experienced, but the manner in which it is experienced, may be important.

Our task was designed to allow participants to learn about the extrinsic uncertainty. We explained the different trial types with a cover story that suggests a model for different kinds of uncertainty across conditions consistently denoted by differently coloured stimuli, and participants received feedback through which they could learn about the uncertainties of the two different cues. Our results showed that there was some learning of the extrinsic uncertainty: participants were not completely blind to it, as would be the case if they had not learned about it. An open question is the time scale over which observers might or might not perfectly learn and/or use this kind of uncertainty during decision-making: given much longer experience with stimuli like these, how much closer would performance come to optimal? Even with perfect learning of the external variability, would people still account for this differently to internal variability in their perceptual decisions? How does this generalize to other tasks and sources of external variability/uncertainty? This calls for longer studies, supported by ideal observer models of the process of learning about uncertainty based on the feedback given.

A key benefit of weighted combination of estimates is a reduction in variance. An unexpected result of the present study is that participants in the intrinsic uncertainty only condition did not reduce their variance, even though they used near-optimal weights. Our model-based analysis excludes the interpretation that they were switching rather than averaging estimates, so we believe that the most likely explanation for the absence of this finding is that of our unplanned mis-matching of reliabilities across intrinsic and intrinsic+extrinsic uncertainty conditions. As with all cue combination studies, attending to this issue is important to reliably detect variance reductions [48,49]. Using continuous response (as compared with alternative forced choice) methods, as here, has advantages for collecting highly informative measurements, but should in future be checked and corrected for central tendency biases in order to accurately recover measurements of variability [44].

## 5. Conclusion

Decision making under uncertainty varies in many ways across real-life settings and experimental tasks. We have highlighted one under-studied but crucial issue: the origin of the uncertainty itself, intrinsic or extrinsic. With a novel perceptual task designed to manipulate this factor alone, we show that while participants account well for intrinsic uncertainty, they under-account for extrinsic. The time scale for learning about this kind of uncertainty, and the generality of this finding for other tasks, settings, and sources of external uncertainty, are open questions for further research.

## Data Availability

All stimuli, data and code are available from the OSF repository: https://osf.io/6paq9/. The data are provided in the electronic supplementary material [51].

## Endnotes

1 Other properties of observers, such as computational limitations, can also distinguish them from ideal observers. For the present discussion, we focus just on the more elementary issue of the noise (variability) affecting observers' estimates.

2 There are some interesting but limited cases in which evolution or (in artificial agents) programming could also provide an ideal observer with this information—mainly for simple physical processes, less obviously for specific cases—e.g. the waiting times on this bus route in particular, the shooting accuracy of this particular archer.

3 We consider localizing the centre of a dot-cloud to be an elementary spatial localization task (similar task to finding the centre of a dim blob [7]). Dot clouds allow presentation of spatially-uncertain stimuli to be operationalized in a highly controllable way, and multisensory combination tasks using dot clouds are well fit by standard cue combination models [20,40].
